# Mechanisms of Regulating Tissue Elongation in *Drosophila* Wing: Impact of Oriented Cell Divisions, Oriented Mechanical Forces, and Reduced Cell Size

**DOI:** 10.1371/journal.pone.0086725

**Published:** 2014-02-04

**Authors:** Yingzi Li, Hammad Naveed, Sema Kachalo, Lisa X. Xu, Jie Liang

**Affiliations:** 1 School of Biomedical Engineering and Med-X Research Institute, Shanghai Jiao Tong University, Shanghai, China; 2 Department of Bioengineering, University of Illinois at Chicago, Chicago, Illinois, United States of America; 3 Shanghai Center for Systems Biomedicine, Shanghai Jiao Tong University, Ministry of Education, Shanghai, China; 4 CAS-MPG Partner Institute for Computational Biology, SIBS, CAS, Shanghai, China; 5 Shanghai Engineering Research Center of Medical Equipment and Technology, Science and Technology Commission of Shanghai Municipality, Shanghai, China; University of Zurich, Switzerland

## Abstract

Regulation of cell growth and cell division plays fundamental roles in tissue morphogenesis. However, the mechanisms of regulating tissue elongation through cell growth and cell division are still not well understood. The wing imaginal disc of *Drosophila* provides a model system that has been widely used to study tissue morphogenesis. Here we use a recently developed two-dimensional cellular model to study the mechanisms of regulating tissue elongation in *Drosophila* wing. We simulate the effects of directional cues on tissue elongation. We also computationally analyze the role of reduced cell size. Our simulation results indicate that oriented cell divisions, oriented mechanical forces, and reduced cell size can all mediate tissue elongation, but they function differently. We show that oriented cell divisions and oriented mechanical forces act as directional cues during tissue elongation. Between these two directional cues, oriented mechanical forces have a stronger influence than oriented cell divisions. In addition, we raise the novel hypothesis that reduced cell size may significantly promote tissue elongation. We find that reduced cell size alone cannot drive tissue elongation. However, when combined with directional cues, such as oriented cell divisions or oriented mechanical forces, reduced cell size can significantly enhance tissue elongation in *Drosophila* wing. Furthermore, our simulation results suggest that reduced cell size has a short-term effect on cell topology by decreasing the frequency of hexagonal cells, which is consistent with experimental observations. Our simulation results suggest that cell divisions without cell growth play essential roles in tissue elongation.

## Introduction

Regulation of cell growth and cell division plays fundamental roles in tissue morphogenesis [1]–[4]. Studies based on model systems such as epithelial cells can help to elucidate mechanisms of controlling tissue formation, organ development, and cancer progression [5]–[8]. *Drosophila* wing imaginal disc, an epithelial sheet of about 50,000 cells that originated from 30 cells within the anlage [9]–[11], is a commonly used model system for studying tissue morphogenesis [12]–[16]. During development, cells in the wing imaginal disc proliferate, forming an elongated tissue shape along its proximal-distal (PD) axis [3], [17], [18]. Although the molecular mechanisms of regulating tissue elongation in *Drosophila* wing have been the subject of extensive studies [19]–[23], the cellular mechanism that dictates this tissue structure is not yet fully understood.

One determinant of tissue elongation is the orientation of cell divisions. Oriented cell divisions can regulate tissue growth along a specific direction in a variety of tissues [2], [3], [17], [18]. A number of molecular players affecting the orientation of cell divisions have been identified. Dachs is a molecule known to mediate the orientation of cell divisions in the developing *Drosophila* wing [18]. When *dachs* is mutated (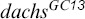), the orientation of mitotic spindles is disrupted, and the division plane is altered, resulting in an adult wing with reduced length along the PD-axis [18]. Theoretical studies suggest that Dachs may indirectly orient the mitotic spindle as a result of the elongated cell shape due to the polarized apical cell junctions [18]. In addition to Dachs, microtubules are another class of molecules that influence the orientation of mitotic spindles during cell divisions [24]–[27]. Microtubules in the cells in *Drosophila* wing align with the PD-axis [28]–[31]. Dachsous (Ds), an atypical cadherin, has also been shown in mutant studies to regulate microtubule organization, as microtubules are less aligned with the PD-axis in *ds* mutants [31]. The orientation of cell divisions is also less focused along the PD-axis in *ds* mutants than in wild-type [17].

Oriented mechanical forces are another determinant of tissue elongation in both plants and animals [4], [32], [33]. A contractile force is exerted by Dachs on apical cell junctions at the distal end of each cell and the proximal end of its neighbor [18]. Cell-cell junctions experience more tension along the PD-axis than in the other directions [18]. In addition, external forces generated by the contraction of the wing hinge are sufficient to induce tissue elongation [17]. The theoretical study further suggests that shear forces are sufficient to drive the PD-axis elongation [17].

Cell size reduction may also contribute to tissue elongation. Between 15 and 24 hour after puparium formation, cells in *Drosophila* wing have reduced cell size after one or two rounds of oriented cell divisions during pupal development. While the wing-blade area remains constant, the shape of the wing becomes elongated along the PD-axis and narrowed along the AP-axis [17].

Although prior studies have demonstrated the sufficiency of either oriented cell divisions or oriented mechanical forces in driving tissue elongation independently [3], [17], [18], less is known about their quantitative effects and their relative contributions. Given the now well established facts that cell proliferation does not equal cell growth, and increased cell proliferation can result in reduced cell size [34], [35], how cell size reduction affects tissue elongation is largely unknown. In addition, understanding how these factors are integrated and collectively determine tissue elongation remains a challenging problem.

Here we use a recently developed two-dimensional cellular model to study the mechanisms of regulating tissue elongation in *Drosophila* wing [36]–[38]. We examine the effects of oriented cell divisions, oriented mechanical forces, as well as reduced cell size on tissue elongation. Our simulation results show that oriented cell divisions and oriented mechanical forces act as directional cues during tissue elongation. Between these two directional cues, oriented mechanical forces have a stronger influence. Our simulation results also reveal a novel mechanism of reduced cell size in promoting tissue elongation. We find that reduced cell size alone cannot drive tissue elongation, as it does not have directional information. However, when combined with directional cues such as that from oriented cell divisions or oriented mechanical forces, reduced cell size can greatly enhance tissue elongation in *Drosophila* wing. Furthermore, our simulation results show that reduced cell size has a short term effect on cell topology. We hypothesize that cell divisions without cell growth play essential roles during tissue elongation in *Drosophila* wing.

## Methods

### Geometry and Mechanics of Cellular Model

**Figure 1:**
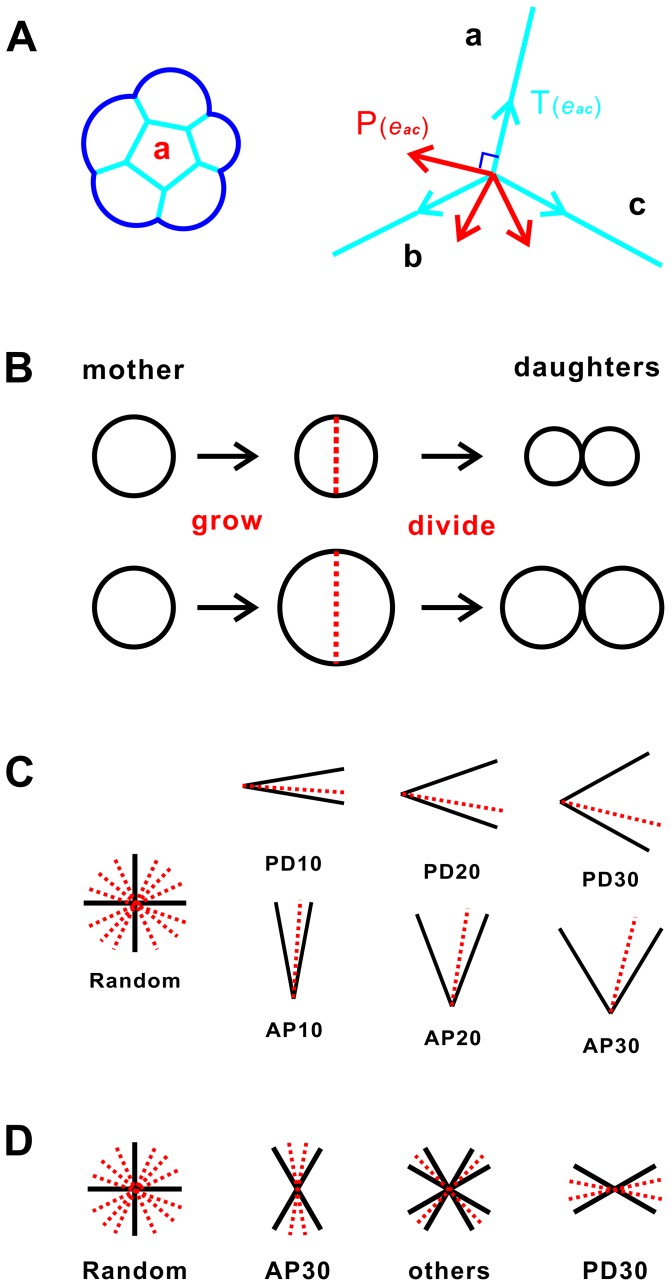
Simulation methodology of cellular model. (A) Left, cells are presented by geometric elements of cell, edge, and vertex. Right, mechanical forces are modeled as tension (blue) and pressure (red). (B) Growth model of reduced cell size (RCS) and non-RCS. In the RCS model, cells proliferate but do not grow. In the non-RCS model, cells grow and proliferate. (C) Division model of oriented cell divisions (OCD) and non-OCD. In the OCD model, the division plane is chosen from uniform distributions of angles in [−10°, 10°], [−20°, 20°], and [−30°, 30°], with respect to the PD-axis and the AP-axis, respectively. (D) Models for oriented mechanical forces (OMF) and non-OMF. In OMF models, tension coefficient *η* is set to 0.75, 1.0, and 1.5, when a cell edge is within [0°, 30°] (PD30), [30°, 60°] (others), and [60°, 90°] (AP30) with respect to the PD-axis, respectively.

We use a recently developed cellular model [38] to study the mechanisms of regulating tissue elongation in *Drosophila* wing. This model captures both geometric properties of cells, including area, length, and internal angles (Fig. 1A), and key cellular mechanical forces.

We provide a brief summary of the cellular model below (details can be found in [38]). An epithelial cell is represented by an 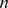-sided polygon when surrounded by 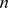 neighboring cells, and has circular free boundaries when not in contact with other cells [36]–[38]. The mechanical forces in a cell are represented as *tension* and *pressure* (Fig. 1A): Tension represents compression forces acting on a cell. It originates from cytoskeletal microfilaments [39]–[41], intermediate filaments [42], and cell membrane [43]. In addition, there exists adhesion [44]–[46] or alternatively repulsion force [47] between cells. These forces can be summed up and modeled as a tension force that exerts along the direction 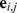 of an *inner edge* (interior cell boundary represented as a straight line segment), or along the tangent direction of an *outer edge* 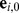 (free cell boundary represented as an arc or a circle). Pressure represents the expansion forces. It arises mostly from microtubules [40], [41], [48], [49] and extracellular matrix [43], [50].

For an edge 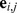, which can be either an inner edge or an outer edge, the tension force is always tangential to the edge 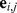:
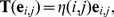



where 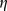 is the tension coefficient, which may depend on the cell types, and 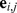 is the unit vector in the direction of shortening 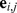. When 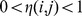, there is a strong adhesion force. When 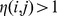, the adhesion force is weak.

For an inner edge 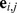 between cells 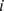 and 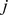, the net pressure force is proportional to the difference in pressure in cell *i* and *j*. It is in the direction normal to the edge 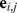, from the cell with higher pressure to the cell with lower pressure. For an outer edge 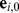 of cell 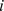, the value of the pressure inside cell 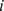 is determined by the curvature of the edge.

### Model for Reduced Cell Size

We simulate the effect of reduced cell size (RCS) between 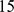 and 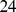 hour after puparium formation during pupal development following reference [17]. A previous study suggests that cell growth disturbs cell shapes in a random fashion such that the atypical myosin Dachs at times is unable to orient every cell to elongate and divide along the PD-axis [18]. To study this effect, we examine two different schemes of cell growth: *non-RCS* and *RCS* (Fig. 1B).

#### non-RCS

Cells grow during cell proliferation. When cells reach the predefined preferred cell size, they become mitotic cells. Each daughter cell after cell division inherits approximately half of the size of the mother cell. This scenario is a typical computational approximation to mimic normal growing cells, which is widely used in cellular models [17], [51]–[54].

#### RCS

Cells do not grow during cell proliferation. Individual cells are randomly chosen as mitotic cells. Each daughter cell inherits approximately half of the size of the mother cell. This scenario is used to model the effects of reduced cell size observed between 15 and 24 hour after puparium formation during pupal development [17].

### Model for Cell Division Plane

We also simulate the effect of oriented cell divisions (OCD), which has been observed between 15 and 24 hour after puparium formation during pupal development [17]. We examine tissue elongation under different schemes of cell divisions (Fig. 1C).

#### non-OCD

The angle of the division plane is randomly chosen from a uniform distribution of all angles. This scheme models the scenario that the effect of the orientation of division plane is insignificant for tissue elongation.

#### OCD

The division plane is chosen from uniform distributions of angles in three different intervals of [−10°, 10°], [−20°, 20°], and [−30°, 30°], with respect to the PD-axis and the AP-axis, respectively. This scheme models the scenarios that division planes orient at specific angles and may influence tissue elongation.

### Model for Oriented Mechanical Forces

To simulate the effect of the oriented mechanical forces (OMF) observed between 15 and 24 hour after puparium formation during pupal development [17], we examine different schemes of mechanical forces exerting on cell edges.

#### non-OMF

Mechanical forces on all edges are of the same magnitude. Tension coefficients 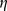 on all edges are set to 1.

#### OMF

Mechanical forces are different according to the angles of cell edge. Tension coefficients 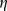 are set to 0.75, 1.0, and 1.5, respectively, when the angles of cell edge are distributed within the range of [0°, 30°] (PD30), [30°, 60°] (others), and [60°, 90°] (AP30) with respect to the PD-axis. This mimics the experimental observations that mechanical forces are doubled on cell boundaries lying at angles close to the AP-axis compared to those on cell boundaries lying at angles close to the PD-axis [18].

### Quantification of Tissue Shape and Tissue Elongation

We approximate the PD-axis and AP-axis in *Drosophila* wing with the direction of 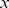-axis and 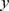-axis, respectively. We define the *tissue shape index* 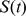 at time 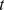 based on the lengths of the tissue along the PD-axis and the AP-axis:
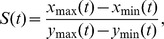



where 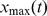 and 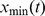 are the maximal and minimal coordinates of the tissue along the 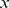-axis (the PD-axis), *respectively*, and 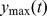 and 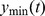 are the maximal and minimal coordinates of the tissue along the 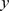-axis (the AP-axis), respectively.

We define the *tissue elongation index* 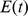 along the PD-axis at time 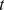 based on the ratio of tissue shape index 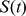:
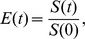



where 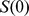 and 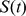 are the tissue shape indices at the beginning and at time 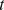 of the simulations, respectively.

### Simulation Methodology

#### Generating samples of initial tissue

Our initial condition is a single cell. Cells grow with time and divide until the tissue contains about 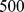 cells. Mitotic cells are chosen as cells whose volume exceed a threshold, and are divided into two daughter cells with approximately equal volume. When cells divide, the largest cell side is chosen for placement of the division plane. This division scheme can produce topological distributions of cells as observed in *Drosophila* wing [36], [38]. We repeat our simulations 30 times, each resulting in an initial random sample of about 500 cells. The resulting tissues of about 500 cells are isotropic and are not elongated.

#### Tissue elongation

We found that tissue elongation index is independent of the shape of initial tissue samples (Figures S1, S2 in File S1). For clarity, we therefore discuss studies of tissue elongation using a tissue of about 500 cells obtained from the initial random sample simulation. We divide cells for 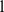 to 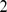 generations (as found in [17]), until the tissue reaches about 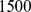 cells, which mimics the pupal development between 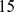 and 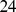 hour after puparium formation. Tissue elongation is simulated with combinations of different growth models, division models, and force models as described in Section 2.2–2.4. We examine the tissue elongation index 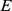 during development. For each combination of different model choices, we run simulations for 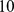 times and take the average as our results.

Our model is implemented in C++. Simulations were performed with 64-bit Linux cluster.

## Results

### Oriented Cell Divisions Drive Tissue Elongation to a Limited Extent

We first computationally studied the effect of oriented cell divisions (OCD) with our cellular model to mimic the pupal development between 15 and 24 hour after puparium formation in *Drosophila* wing, without considering the effects of reduced cell size and oriented mechanical forces. The orientation of division plane is chosen from three uniform distributions, with orientation angles with respect to the PD-axis and the AP-axis (*e.g.*, PD10) in intervals of [−10°, 10°], [−20°, 20°], and [−30°, 30°], respectively. Results are compared with those obtained using random division choice.

**Figure 2:**
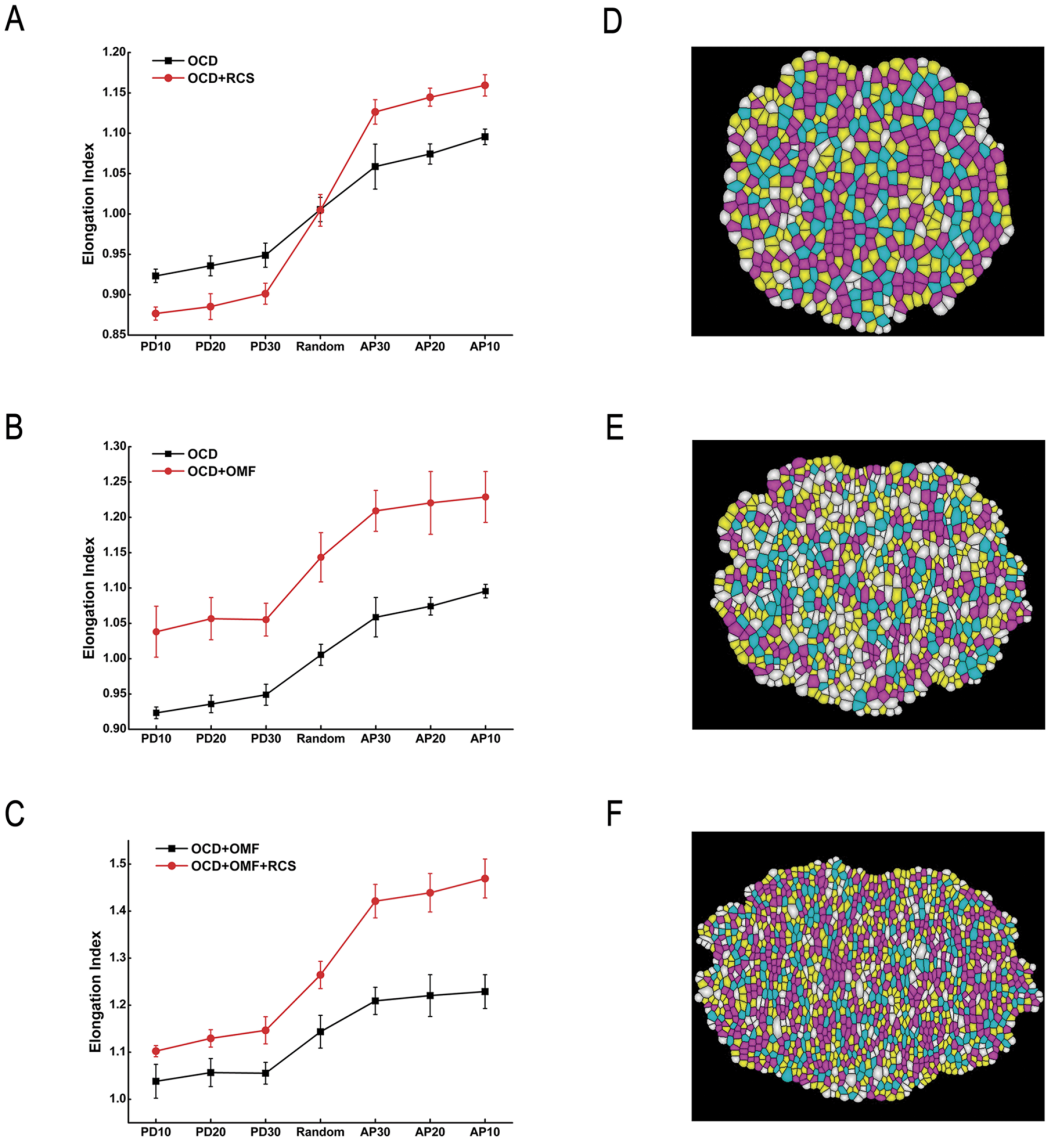
Simulation results of tissue elongation. The elongation index is plotted against orientation angle for different cell models. (A) Oriented cell divisions drive tissue elongation, but only to a limited extent (black). Reduced cell size, when combined with oriented cell divisions, enhances tissue elongation (red). (B) Oriented mechanical forces produce significant tissue elongation along PD-axis. (C) Reduced cell size significantly enhances tissue elongation when both directional cues are present. (D–F) Morphology at the beginning, midpoint, and the end of the simulation with oriented cell division (AP10), oriented mechanical forces, and reduced cell size.

We found that without oriented cell divisions, tissue elongation is absent throughout the simulation. The tissue elongation index 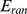 at the end of the simulation is 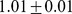 when random division is chosen (Fig. 2A), reflecting the fact that tissue shapes at the beginning and the end of the simulation are similar.

**Figure 3:**
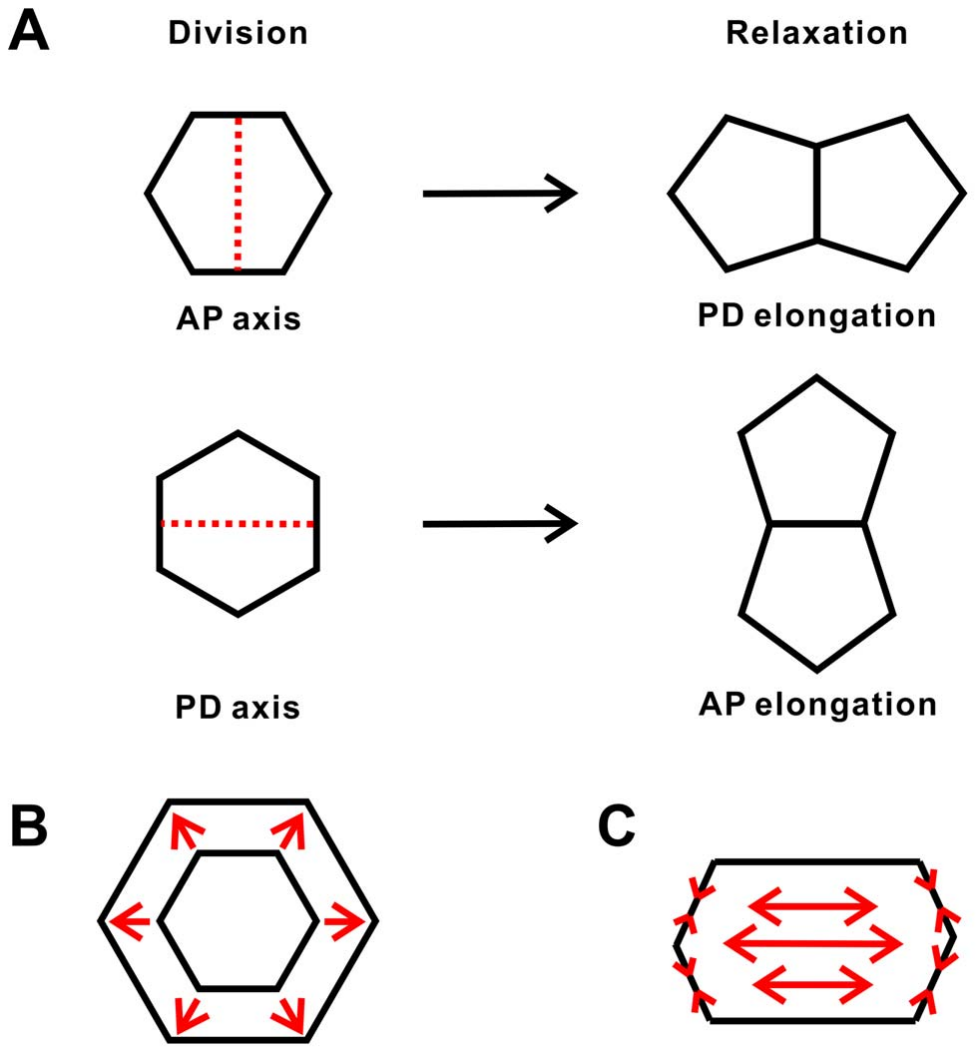
Physical illustrations of different simulation choices. (A) AP-axis division leads cells to elongate in PD-axis (upper), and PD-axis division leads cells to elongate in AP-axis (lower). (B) Isotropic cell growth makes cells grow and move in all directions, and reduced cell size constraint cells to move in the direction of tissue elongation. (C) Oriented mechanical forces lead the shape of cells to change in oriented directions.

In contrast, with oriented cell divisions, we can generate elongated tissue shapes along different directions, although only to a modest extent. Specifically, if cells are divided along AP-axis, tissue will elongate along PD-axis (Fig. 3A). The tissue elongation index 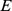 with division choices along AP-axis are 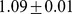, 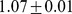, and 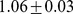, when the orientation angles fall into the intervals of AP10, AP20, and AP30, respectively (Fig. 2A). Similarly, if cells divide along PD-axis, tissue will elongate along AP-axis, and will shorten along PD-axis (Fig. 3A). The tissue elongation index 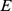 when divisions are along PD-axis with angles falling into the intervals of PD10, PD20, and PD30 are 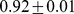, 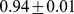, and 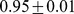, respectively (Fig. 2A).

Oriented cell divisions can only drive tissue elongation to a limited extent, as the elongation index is still small, *e.g.*, 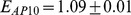. We found that the degree of tissue elongation is influenced by the intervals of the angles from which the division plane is chosen. The smaller the interval around the AP or PD axis, the higher the degree of tissue elongation is. With oriented cell divisions alone, we cannot reproduce the elongated tissue shape to the extent observed in observed in experiments between 15 to 24 hour after puparium formation [17], indicating that other factors also influence tissue elongation during pupal development. Overall, our simulation results suggest that oriented cell divisions serve as directional cues to tissue elongation and they do not work alone.

### Reduced Cell Size Enhances Tissue Elongation in Combination with Oriented Cell Divisions

In this section, we simulate the effect of reduced cell size (RCS) on tissue elongation in conjunction with oriented cell divisions. Here we do not take into consideration the effects of oriented mechanical forces. This section of our simulation reflects the fact that cells between 15 and 24 hour after puparium formation do not grow during pupal development [17].

We found that reduced cell size alone does not affect tissue shape when cell division planes are randomly oriented. Tissue elongation index with reduced cell size 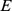 (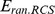) is 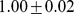, non-distinguishable from that without reduced cell size (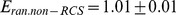) (Fig. 2A). Our simulation results indicate that reduced cell size itself does not provide directional cues for tissue elongation. It has no effect on the change of tissue shape without other directional cues.

However, reduced cell size can significantly amplify the effect of directional cues provided by oriented cell divisions in changing tissue shape. With non-RCS growth, tissue elongation index 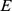 with division plane oriented along the AP-axis are 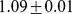, 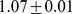, and 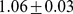, respectively, when the orientation angle falls into the intervals of AP10, AP20, and AP30, respectively. With reduced cell size, tissue elongation index 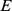 increases significantly to 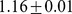, 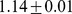, and 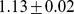, respectively (Fig. 2A). Similarly, with non-RCS growth, tissue elongation index 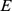 with division plane oriented along the PD-axis are 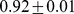, 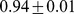, and 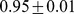, respectively, when the orientation angle falls into the intervals of PD10, PD20, and PD30, respectively. With reduced cell size, tissue elongation index 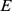 decreases to 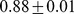, 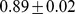, and 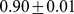, respectively (Fig. 2A). This demonstrates that reduced cell size can significantly enhance tissue elongation when directional cues are provided by oriented cell divisions.

In summary, our simulation results show that reduced cell size has no direct effect on tissue elongation when the orientation of cell division is random. However, division with reduced cell size can promote tissue elongation with the presence of directional cues. While cell growth and cell division both may occur during cell proliferation, isotropic cell growth only results in proliferating cells moving randomly in all directions, with the tissue taking a round shape (Fig. 3B). Cell divisions without cell growth may act to counter the effects of isotropic cell growth and constrain tissue to elongate following the directional cues.

### Oriented Mechanical Forces Significantly Influence Tissue Elongation

Although a combination of oriented cell divisions and reduced cell size can generate elongated tissue shape (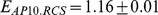), the elongation effect is not as pronounced as observed in experiments between 15 to 24 hour after puparium formation (E =  ca. 1.4) [17]. Oriented mechanical forces (OMF) can also influence tissue elongation. To simulate the effects of oriented mechanical forces, we set the tension coefficients 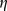 to 0.75, 1.0, and 1.5, respectively, when the angles of cell edge are distributed within the range of [0°, 30°], [30°, 60°], and [60°, 90°] with respect to the PD-axis.

Our simulation results suggest that oriented mechanical forces can generate tissue elongation even with random division orientation. The tissue elongation index with oriented mechanical forces (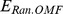) is 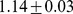 (Fig. 2B). Clearly, oriented mechanical forces can provide directional cues for tissue elongation. It is also interesting to note that oriented mechanical forces have more influence on tissue elongation than oriented cell divisions (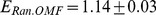 vs 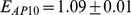).

We then combined both the directional cues, *i.e.*, oriented cell divisions and oriented mechanical forces, under two different scenarios. First, both directional cues influence tissue elongation in the same direction. Second, two directional cues influence tissue elongation in the opposite directions. In the first scenario, tissue elongation index (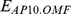) is 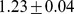 (Fig. 2B), significantly elevated compared to the tissue elongation index 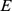 with either of the directional cues (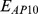  = 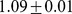 and 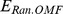  = 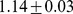). Our simulation results show that oriented mechanical forces and AP-axis cell division work collectively to drive more efficient tissue elongation. In the second scenario, tissue elongation index 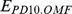 is 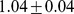 (Fig. 2B). The elongation effect is diminished compared to elongation index (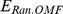  = 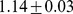) with oriented mechanical forces alone. However, tissue still elongated along PD-axis, the same direction as it was influenced by that of the oriented mechanical forces. We note that the PD-axis cell division drives tissue elongation in the opposite direction (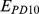  = 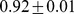). Our simulation results show that oriented mechanical forces are the dominant driving force and can overcome the effect of PD-axis cell division. That is, oriented mechanical forces have stronger influence on tissue elongation than oriented cell divisions.

### Enhancement of Tissue Elongation by Reduced Cell Size Is Determined by Each Directional Cue

We now simulate the effect of reduced cell size in conjunction of both directional cues. We found that reduced cell size can enhance tissue elongation when compared to models with regular cell growth (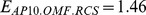 vs 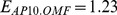, and 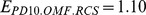 vs 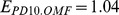) (Fig. 2C). The enhancements of tissue elongation by reduced cell size are different under different conditions as described in Section 3.3. For illustration, we use the ratio of elongation index 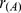 to describe the enhancement of tissue elongation due to reduced cell size in conjunction of different directional cues. 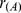 is defined as:
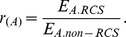



Here 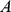 is the directional cue. 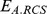 and 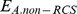 are the elongation index under 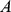 with reduced cell size, and under 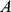 without reduced cell size, respectively.

The enhancement of tissue elongation under oriented mechanical forces by reduced cell size 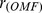 is 1.11 (>1) (Table. 1). In the first case where division planes are along the AP axis, the enhancement on all AP-axis cell divisions is about 1.06 (>1). This indicates the enhancement of reduced cell size on both oriented mechanical forces and AP-axis cell divisions are in the same direction (PD-axis). The enhancement of reduced cell size to the combined cues of AP-axis cell divisions orientation and oriented mechanical forces is 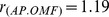 (Table. 1). It is similar to the multiplication of the enhancement to each directional cue (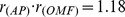) (Table. 1). In the second case where division planes are along the PD axis, the enhancement on all PD-axis cell divisions is about 0.96 (<1). This indicates the enhancement of reduced cell size on oriented mechanical forces and PD-axis cell divisions are in the opposite directions (PD-axis vs AP-axis). Similarly, the enhancement on the combination of oriented mechanical forces and PD-axis cell divisions is similar to the multiplication of the enhancement on each directional cue (Table. 1). Our simulation results suggest that the enhancement of tissue elongation by reduced cell size on combined directional cues is determined by the enhancement on each directional cue.

**Table 1:** Enhancement of tissue elongation by reduced cell size.

OMF		AP divisions + OMF		PD divisions + OMF	
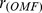	1.11	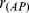	1.06	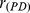	0.96
		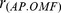	*1.19*	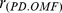	*1.06*
		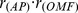	*1.18*	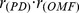	*1.06*

### Comparison to Experimental Observations

Our simulation results show that oriented cell divisions and oriented mechanical forces both serve as directional cues to drive tissue elongation. Reduced cell size can significantly enhance tissue elongation when combined with these directional cues. We hypothesize that oriented cell divisions, oriented mechanical forces, and reduced cell size work collectively to regulate tissue elongation in *Drosophila* wing between 15 to 24 hour after puparium formation.

We compared our simulation results with the experimental work in reference [17]. We examined the change of tissue shape exactly between 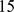 and 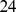 hour after puparium formation during pupal development. Tissue elongation index 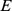 from 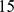 to 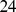 hour after puparium formation was approximately 1.40 in reference [17]. Simulation results in our model showed that 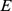 is greater than 1.40 only when we combined the choices of oriented cell divisions, oriented mechanical forces, and reduced cell size (1.42, 1,44, and 1.46 respectively with AP30, AP 20, and AP10 division choices). Morphology at the beginning, midpoint, and the end of the simulation with combined choices of oriented cell division (AP10), reduced cell size, and oriented mechanical forces are shown in (Fig. 2D–F). Other combinations of growth choice, force choice, and division choice can only generate a tissue elongation index 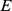 of 1.26 or less. Our simulation results suggest that oriented cell divisions, oriented mechanical forces, and reduced cell size can all mediate tissue elongation, although they are functionally different.

## Discussion

### Effect of Oriented Cell Divisions

Our simulation results show that oriented cell divisions can drive tissue elongation. Molecules such as Ds influence the orientation of planar cell polarity during development in *Drosophila* wing. It is possible that oriented cell divisions help to maintain some of the initial polarity pattern when forming new cell boundaries. After oriented cell divisions, cells relax, and the tissue elongates in a specific direction (Fig. 3A). However, oriented cell divisions alone are not sufficient to reproduce the experimental observations. Oriented cell divisions likely work together with other directional cues, such as oriented mechanical forces, to drive tissue elongation. In the work by Mao *et al*., isometric tension with oriented cell division produced more elongated and PD aligned tissue than *in vivo* clones [18]. The difference between their model and ours may be due to the different model parameters. In Mao *et al*., the division plane was always exactly perpendicular to the PD-axis. However, their experimental results showed that the orientation of cell divisions was imperfectly correlated with the PD-axis in *Drosophila* wing disc in culture [18]. The division plane in our simulation is set in a specific range around PD-axis according to experimental observations [17]. Moreover, the simulations by Mao *et al*. were run for around 4 to 5 generations (48 hours) [18], while in our simulations, cells are divided only for 1 to 2 generations, which tracks more realistically the pupal development between 15 to 24 hour after puparium formation [17].

Our simulation results also suggest oriented cell divisions alone have limited effects on tissue elongation. Oriented cell divisions may contribute to tissue elongation via two distinct mechanisms: (1) tissue elongates by oriented cell divisions while cell growth is isotropic; (2) cell growth is anisotropic, and cell divisions with orientation along the longest axis reduce the stress exerting on cell boundaries so that regular cell shape forms [55]. With oriented cell divisions alone, we cannot reproduce the elongated tissue shape observed in experiments. However, with combined oriented cell divisions, oriented mechanical forces, and reduced cell size, we are able to reproduce tissue elongation observed in *Drosophila* wing qualitatively [17]. Oriented mechanical forces in our model can have an equivalent effect as that of anisotropic cell growth.

### Effect of Oriented Mechanical Forces

Our simulation results show that oriented mechanical forces have stronger effects on tissue elongation than the oriented cell divisions. Our simulation results also suggest that oriented mechanical forces, oriented cell divisions, and reduced cell size might work collectively to influence tissue elongation in *Drosophila* wing between 15 to 24 hour after puparium formation. In the work by Mao *et al*., oriented tension and division along the long axis were sufficient to drive tissue elongation [18]. Their results agree with ours to a certain extent, although there is no consideration of the effects of reduced cell size. Dachs, producing oriented mechanical forces at the apical junctions, can indirectly orient cell divisions. Oriented mechanical forces result in cell shape elongated along the PD-axis prior to cell divisions, which then orient the mitotic spindles. At the same time, mitotic spindles always align along the long axis of cells in mammalia [56]. Thus the strategy of oriented tension and division along the long axis in Mao's study seems to be equivalent to combining oriented mechanical forces and oriented cell divisions in our model. Mao *et al*. did not take into consideration of reduced cell size in their model, because in their study, they were modeling a different development stage. Altogether, the stronger influence of oriented mechanical forces on tissue elongation *vs* oriented cell divisions is demonstrated.

In addition to the Dachs myosin, two large and atypical cadherins, Ds and Fat, are involved in planar cell polarity (PCP) pathways of *Drosophila* wing [3], [57]. Mediating cell-cell interactions through adhesion is an important function of cadherin [57]. Mechanical forces by atypical cadherins with polarized properties can help to maintain cell polarity and regulate cell proliferation so that they contribute to different aspects of tissue morphogenesis such as shape and size.

### Effect of Reduced Cell Size

Our simulation results show that reduced cell size alone cannot drive tissue elongation. However, when combined with directional cues such as oriented cell divisions or oriented mechanical forces, reduced cell size can significantly enhance tissue elongation in *Drosophila* wing. We suggest that tissue shapes are not affected by isotropic cell growth as cells are moving randomly, and cell divisions without cell growth may act to counter the effects of isotropic cell growth and constrain tissue to elongate following the directional cues. Reduced cell size can affect tissue elongation through differences in mechanical forces. It was shown recently that “mechanical relaxation” has significant effects in tissue pattern formation during tissue growth [58]. Variation in mechanical relaxation ultimately affects tissue structure and tissue shape. Our previous study also showed that growing cells reach the mechanical equilibrium and equilibrium tissue structure only after certain time duration [36]. In the current study, cell proliferation without growth, namely, reduced cell size, may constrain “mechanical relaxation” when cells divide without growing, which may lead to more elongated tissue shape. Our hypothesis can be verified experimentally by treating the tissue with inhibitor of cell division, such as Y-27632 [59]. According to findings of this study, we predict that there will be less tissue elongation after such treatment, as there will be more time for mechanical relaxation.

The overall interplay of cell size, cell division, tissue shape, and tissue structure has been a long standing problem in developmental biology [60]. The effect of cell size on proliferation and organ size has been recently observed [61]. In that study, the authors identified a gene *fo* that would change the number of cells and the size of each cell in the petal of *Antirrhinum* upon mutation. The number of cells was significantly increased, while the size of each cell was significantly decreased. These results suggested a compensatory mechanism between cell size and the number of cells for maintaining wild-type organ size. Our simulation results suggest that the cell size may also play an important role in maintaining wild-type organ shape. It is well known that morphogen gradients such as Dpp maintain tissue shape through oriented cell divisions, which result in anisotropic growth [62]. It is possible that by reducing their size, cells sense a different morphogen gradient and this can affect oriented cell divisions. It would be interesting to quantify such effect in future simulation studies, where the oriented cell divisions can be linked to morphogen gradients. Overall, it is likely that cell division without cell growth may play important roles in affecting cell mechanics and in influencing how cells sense and respond to morphogen gradient, therefore affecting formation of specific tissue and organ shape.

### Cell Topology during Tissue Elongation

Our previous studies suggest that the orientation of division plane and mechanical forces play important roles on regulating cell topology [36]–[38]. It is likely that oriented cell divisions, oriented mechanical forces, and reduced cell size may also affect cell topology during tissue elongation. To address this issue, we examined hexagonal frequencies with different combinations of growth choices, division choices, and force choices.

#### Effect of oriented cell divisions and oriented mechanical forces

**Figure 4:**
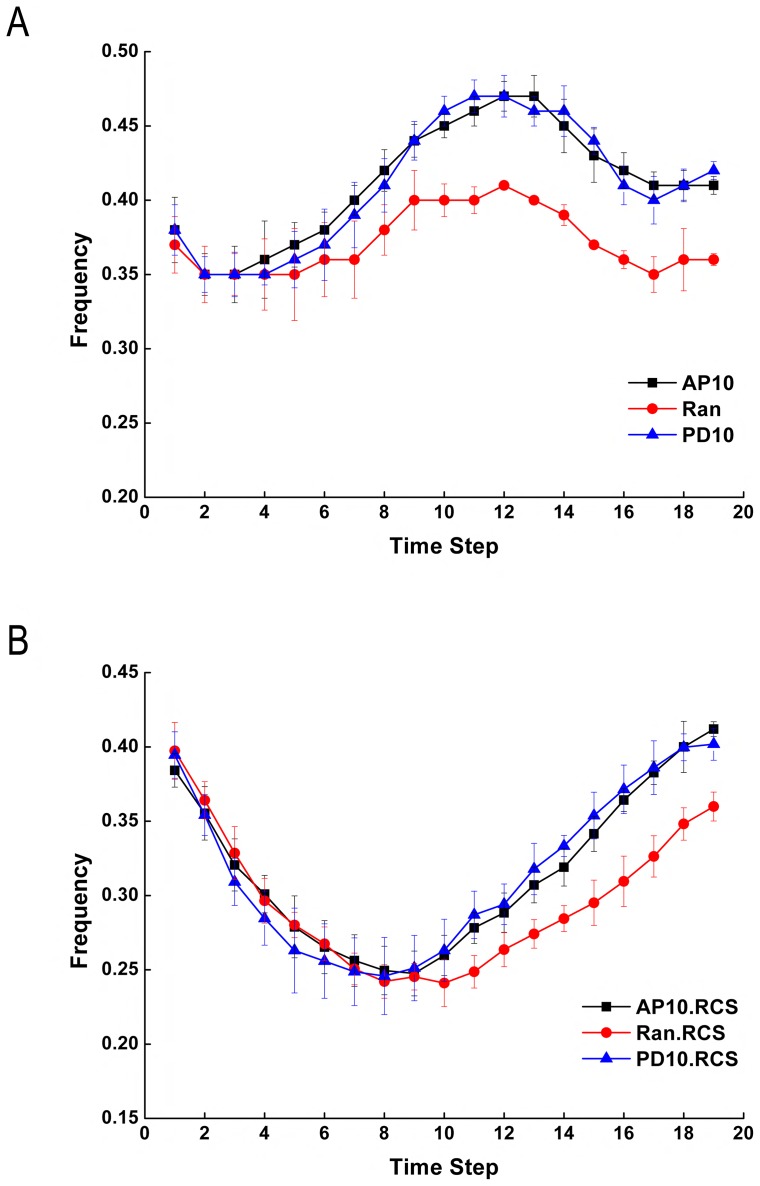
Hexagonal frequencies during tissue elongation by different simulation choices. (A) Oriented cell divisions increase the hexagonal frequencies compared with the random division choice. (B) Reduced cell size has a short term effect of decreasing the hexagonal frequencies.

We found that oriented cell divisions produced more hexagonal cells compared with the random division choice. Without the consideration of oriented mechanical forces and reduced cell size, the hexagonal frequencies were the same for all three scenarios (Ran, AP10, and PD10) at the beginning of the simulation. After certain time steps, oriented cell divisions (AP10 and PD10) generated higher hexagonal frequencies (by about 0.05) compared with the random division choice, and this higher hexagonal frequency is maintained afterwards (Fig. 4A). Higher hexagonal frequency by oriented cell divisions was also shown in other scenarios when the oriented mechanical forces and reduced cell size were taken into consideration (Figures S3–S6 in File S1). This suggests that oriented cell divisions can increase the amount of hexagonal cells and can generate more structured tissue pattern compared with the random division choice. Our simulation results also showed that hexagonal frequencies were not affected using the oriented mechanical forces (Figures S4, S6 in File S1). This suggests that oriented mechanical forces have little influence on cell topology during tissue elongation.

#### Effect of reduced cell size

We then took the reduced cell size into consideration. We found that the hexagonal frequencies decreased at the beginning, but increased afterwards (Fig. 4B). The average of hexagonal frequencies over all time steps are different between the two scenarios (RCS vs non-RCS). With RCS, much less hexagonal cells are produced compared with that when non-RCS models are used (Table. 2). However, at the end of the simulation, both scenarios generated similar hexagonal frequencies (Table. 2). This suggests that reduced cell size has a short term effect on cell topology by decreasing the hexagonal frequency. After the tissue relaxes, the effect of decreased hexagonal frequency is lost.

**Table 2:** Comparison of hexagonal frequencies between RCS and non-RCS growth choices.

	AP10		Random		PD10	
Hexagonal frequency	RCS	Non-RCS	RCS	Non-RCS	RCS	Non-RCS
Last time step	0.41	0.41	0.36	0.36	0.40	0.42
Average of all steps	*0.32*	0.41	*0.30*	0.37	*0.32*	0.41

We compared our simulation results with the experimental work in reference [17]. It was observed that the hexagonal frequency decreased first, and then increased after a certain period in the experiments [17]. This is consistent with our simulation results, despite the difference in initial hexagonal frequency. This difference might be a result of different tissue size. Specifically, our simulation starts from about 500 cells, at which the hexagonal frequency is less than that is observed in the experiments. Overall, our simulation results suggest that reduced cell size affects cell topology during tissue elongation.

### Future Applications

#### Cell divisions and mechanical forces in diseases and cancer

We have previously shown that the orientation of division plane and mechanical forces have significant impacts on regulating cell topology during epithelial proliferation with isotropic growth [38]. Here we found that oriented cell divisions and oriented mechanical forces play important roles in tissue elongation in *Drosophila* wing with anisotropic growth. These directional cues are correlated with molecules such as Dachs and Ds involved in PCP pathways [17], [18], [63], [64]. It would be interesting to study the effects of cell divisions and mechanical forces and their relationship with PCP pathways in epithelial diseases [65], [66], especially in cancer progression [67], [68]. In addition, mechanical forces are tightly correlated in cancer invasion and metastasis [69]–[71]. Cell-cell and cell-matrix interactions are shown to alter significantly during cancer progression both theoretically and experimentally [72]–[74]. Fully understanding the underlying mechanisms that are regulated by cell divisions and mechanical forces may possibly provide potential targets for cancer therapy.

#### Long range morphogen gradient

Dachs is essential for oriented mechanical forces and oriented cell divisions, which can affect tissue elongation. It is suggested that planar polarization of Dachs is ultimately oriented by long-range gradients of secreted morphogens from compartment boundaries [18]. It will be interesting to study the fundamental mechanisms of controlling tissue pattern, size and shape by these secreted molecules.

#### Symmetric and asymmetric cell divisions

In this study, we have examined tissues with symmetric cell divisions, where one cell gives rise to two identical daughter cells. It was shown that in the developing organism, asymmetric cell divisions, in which two daughter cells were generated with different cell sizes and cell fates, played a central role [55], [75], [76]. This is an intrinsic property of stem cells [76]–[78], and defects in asymmetric cell divisions can lead to cancer [76], [79], [80]. It is still puzzling to understand many biological phenomena associated with asymmetric cell divisions. How the relative proportions of cell population each with different fates are achieved? How does stochasticity influence cell division and cell differentiation? It will be interesting to use our model to quantitatively study the fundamental mechanisms to coordinate asymmetric cell divisions with other factors such as cell fates and cell positions during development. A preliminary study into the population dynamics of stem cells using our model has generated important insights [81].

## Conclusions

We have used a recently developed two-dimensional cellular model to study the mechanisms of regulating tissue elongation in *Drosophila* wing between 15 to 24 hour after puparium formation. We simulated the effects of directional cues, including oriented cell divisions and oriented mechanical forces, on tissue elongation. We also computationally analyzed the role of reduced cell size. Our simulation results suggest that tissue elongation in *Drosophila* wing is influenced collectively by oriented cell divisions, oriented mechanical forces, and reduced cell size. We show that oriented cell divisions and oriented mechanical forces act as directional cues during tissue elongation. Between them, we find that oriented mechanical forces have a stronger influence compared to oriented cell divisions. In addition, our simulation results reveal a novel mechanism of reduced cell size in promoting tissue elongation. We find that reduced cell size alone cannot drive tissue elongation, as it does not have directional information. However, when combined with directional cues such as oriented cell divisions and oriented mechanical forces, reduced cell size can significantly enhance tissue elongation in *Drosophila* wing between 15 to 24 hour after puparium formation. Furthermore, our simulation results show that reduced cell size affects cell topology during tissue elongation. Overall, our simulation results suggest that cell division without growth is essential for tissue elongation in *Drosophila* wing.

## Supporting Information

File S1 **Combined Supporting Information.** Figure S1 shows the changes in tissue shape index of samples with different initial shapes; Figure S2 shows tissue elongation index is independent of the shape of initial samples; Figure S3–S6 show the comparison of hexagonal frequencies between different strategies during tissue elongation.
(PDF)
