# Supplementary material for: Mechanisms of Regulating Tissue Elongation in Drosophila Wing: Impact of Oriented Cell Divisions, Oriented Mechanical Forces, and Reduced Cell Size
Source: PLoS One. 2014 Feb 4;9(2):e86725. doi: 10.1371/journal.pone.0086725 (PMC3913577; doi:10.1371/journal.pone.0086725)
Supplement: File S1 — Combined Supporting Information. Figure S1 shows the changes in tissue shape index of samples with different initial shapes; Figure S2 shows tissue elongation index is independent of the shape of initial samples; Figure S3–S6 show the comparison of hexagonal frequencies between different strategies during tissue elongation. (PDF) [file pone.0086725.s001.pdf]

File S1. Combined Supporting Information.

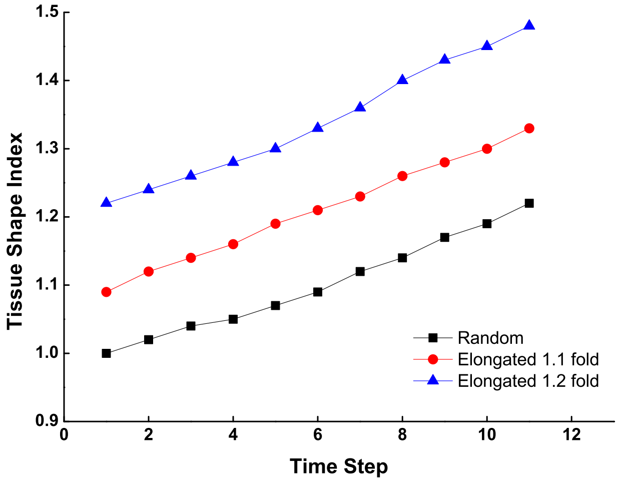

Figure S 1: Changes in tissue shape index of samples with different initial shapes..

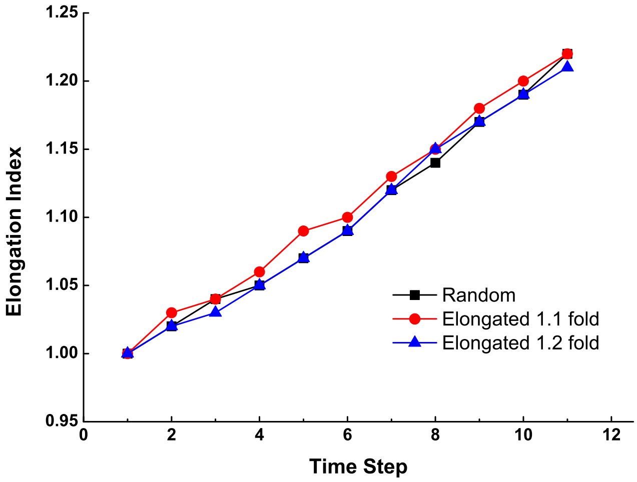

Figure S 2: Tissue elongation index is independent of the shape of initial samples.

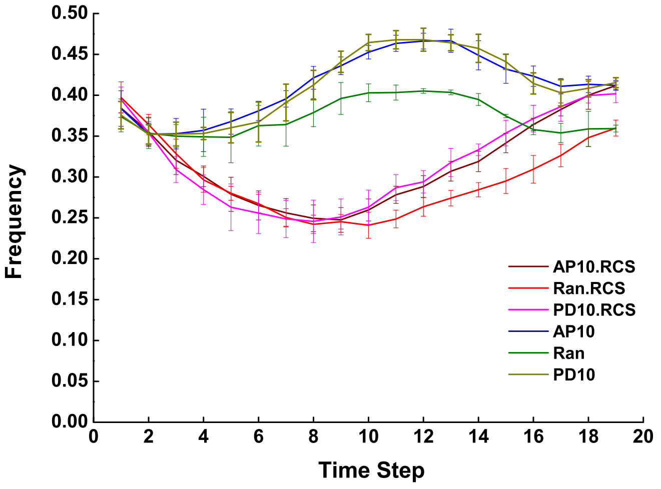

Figure S 3: Comparison of hexagonal frequencies between OCD vs OCD + RCS.

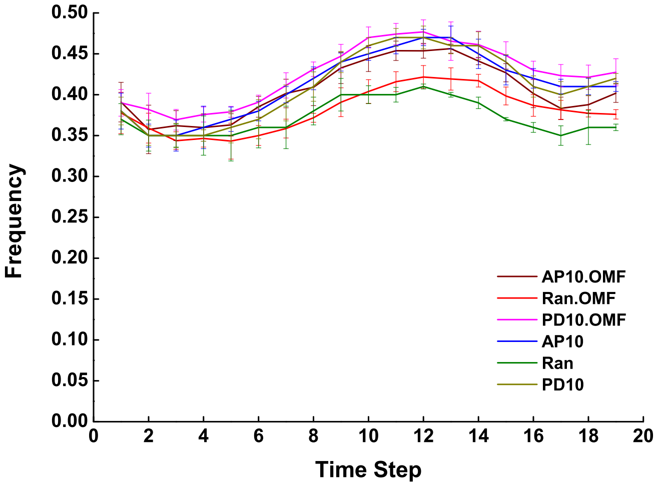

Figure S 4: Comparison of hexagonal frequencies between OCD vs OCD + OMF.

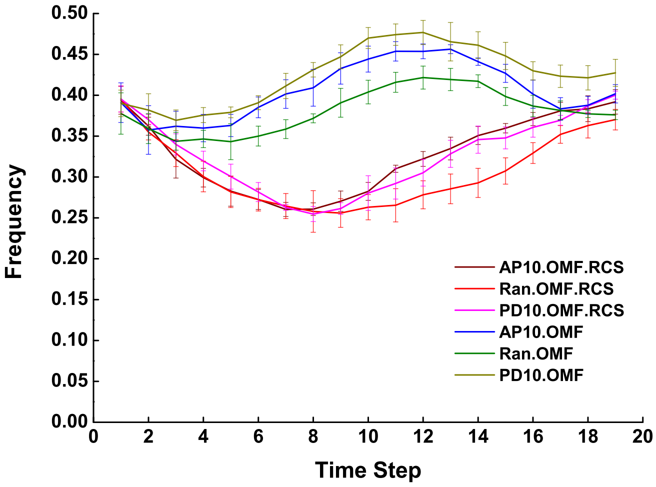

Figure S 5: Comparison of hexagonal frequencies between OCD + OMF vs OCD + OMF + RCS.

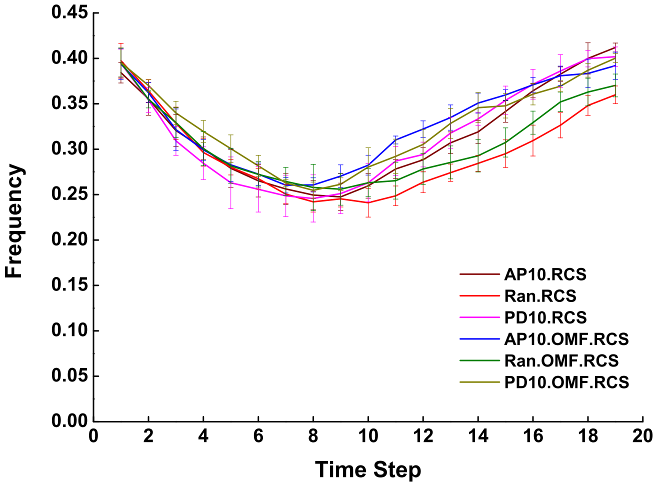

Figure S 6: Comparison of hexagonal frequencies between OCD + RCS vs OCD + OMF + RCS.
